# Effects of eight weeks of mat pilates training on selected hematological parameters and plasma volume variations in healthy active women

**DOI:** 10.1371/journal.pone.0267437

**Published:** 2022-06-03

**Authors:** Nourhen Ghazel, Amine Souissi, Iyed Salhi, Ismail Dergaa, Hugo Cesar Martins-Costa, Sarah Musa, Helmi Ben saad, Abderraouf Ben Abderrahman

**Affiliations:** 1 Higher Institute of Sport and Physical Education of Ksar-Saïd, Manouba, Tunisia; 2 Université de Sousse, Faculté de Médecine de Sousse, Hôpital Farhat HACHED, Laboratoire de Recherche (Insuffisance Cardiaque, LR12SP09), Sousse, Tunisie; 3 PHCC, Primary Health Care Corporation, Preventative Health Department–Wellness, Doha, Qatar; 4 Department of Physical Education, Pontifical Catholic University of Minas Gerais, Belo Horizonte, Minas Gerais, Brazil; Universidade Federal de Mato Grosso do Sul, BRAZIL

## Abstract

**Aim:**

To evaluate the effects of eight weeks of mat Pilates training on selected hematological parameters, ***i*.*e*.** white blood cell, neutrophils, monocyte, lymphocyte, hematocrit, hemoglobin as well as plasma volume variations in healthy, active women.

**Methods:**

Twenty-eight women physical education students volunteered to participate in the present investigation. They were assigned to two groups: a Pilates training group (n = 14) that followed an 8-week Pilates training program, and a control group (n = 14). Blood samples were collected at rest at two separate occasions before and after Pilates training.

**Results:**

The Pilates training group had higher values of plasma volume variations and lower values of white blood cell (19.4%), neutrophils (32%), hematocrit (4.3%) and hemoglobin (4.6%) compared to control group (p<0.05).

**Conclusion:**

The results of the present study suggested that Pilates training could be an effective strategy for increasing plasma volume variations and boosting immune system in healthy active women.

## Introduction

Joseph Pilates developed Pilates using six key principles: centering, concentration, control, precision, flow, and breath **[1–4]**. Pilates training (PT) is intended to improve general body flexibility and health by emphasizing core strength, posture, and coordination of breathing with movement **[4, 5]**. Otherwise, PT has been used to reduce pain and disability **[6]**, to improve sports performance **[2, 4]**, and to treat arterial-hypertension, chronic neck pain, and post-menopausal osteoporosis **[6]**. Some clinical trials chose this non-pharmacological treatment for 12 weeks as a therapy for acute chronic neck pain **[7]**, to enhance functional and cognitive performance in elderly people **[8]**, to increase lower limb strength in Parkinson’s disease patients **[9]**, and to reduce heart rate and creatinine kinase activity in patients **[10]**. Suna et al. **[11]** reported that PT helped sedentary women lose weight faster, increase mobility, and lower resting-maximal heart rate and blood glucose. Pilates has also been used to enhance the aerobic and anaerobic power of multiple sclerosis patients, as well as their endurance and decrease their fatigue frequency **[12]**.

The impact of PT on hematological and biochemical variables has recently become a new field of investigation. It has been demonstrated that PT has a beneficial impact on adipokine levels in overweight women when used for 12 weeks, three days per week for 60 minutes **[13]**, as well as serum levels of certain inflammatory markers such as C reactive protein in sedentary women with overweight **[14]**. Researchers have also shown that PT is a suggested physical activity to promote health and boost the immune system **[15, 16]**. Gronesova et al. **[16]** reported that 40 weeks were effective in improving natural killer immune cells response and inflammatory milieu in plasma of healthy women **[16]**. Moreover, Bahram et al. **[15]** discovered that 12 weeks of PT increased monocyte level and decreased neutrophils count.

Hematological parameters (***e*.*g*.** hemoglobin and hematocrit) and plasma volume variation (PVV) may be considered as a valuable marker in the evaluation of aerobic capacity development **[17–19]**. Indeed, the increase of PVV is associated with an increase of aerobic performances (***e*.*g*.** maximal oxygen uptake) **[18, 19]**. Otherwise, it was found that PT is as effective as aerobic training to improve the aerobic performance **[20]**. It would be therefore interesting to investigate the effects of mat PT on PVV and hematological parameters.

We hypothesized that PT would increase PVV and boost the immune system in active healthy women. To the authors’ finest knowledge, no previous study has examined the effects of PT on PVV and hematological parameters despite the role of plasma as a key transporter of nutriments and oxygen to active muscles **[21]**. Thus, the aim of this study was to investigate the effects of PT on PVV as well as selected hematological parameters in active healthy women.

## Methods

### Participants

Women physical education students (who participate in a variety of activities) having normal menstrual cycle lengths of 26–34 days and do not take oral contraceptives, participated in the study. All the participants were non-smokers, non-sedentary, not pregnant, and they refrained from exercise and alcohol- and caffeine-containing drinks for at least 24 h before the test. Participants were assigned at random to one of two groups: a Pilates training group (PG) that followed an 8-week PT program, or a control group (CG). Trial participants were allocated to the training or control using simple random allocation by the main investigator (*ABA in the authors’ list*). The participants’ anthropometric data were given in **Table 1**.

**Table 1:** Characteristics of participants.

	PG (n = 14)	CG (n = 14)
**Age (years)**	20.57±0.8	20.92±0.9
**Body mass (kg)**	60.74±9.5	63.21±11.8
**Body height (cm)**	165±07	164±6
**BMI (kg.m** ^**2**^ **)**	22.30±2.8	23.02±4.13
**FFM (kg)**	13.06±1.1	13.07±1.2

Data were mean±SD

**BMI**: body mass index, **CG**: control group, **FFM**: fat free mass, **PG**: Pilates training group.

After being informed about all research protocol, each participant gave written informed consent to participate in this study. The study was conducted in accordance with the Declaration of Helsinki guidelines, and was approved by the Ethics Committee of the medical unit of the Institute of Physical and Sports Education in Ksar Said (Tunis, Tunisia).

### Sample size

The sample size was estimated using this formula: N = ((r+1) (Z_α/2_ + Z_1-β_)^2^ σ^2^)/(rd^2^) **[22]**, where:

- “**Z**_**α/2**_” is the normal deviate at a level of significance = 2.58 (1% level of significance);
- “**Z**_**1-β**_” is the normal deviate at 1-β% power with β% of type II error (0.84 at 80% statistical power);
- “**r**” (= n_1_/n_2_, n_1_ and n_2_ are the sample sizes for the PG and CG such N = n_1_ + n_2_) is the ratio of sample size required for two groups (r = 1 gives the sample size distribution as 1:1 for two groups);
- “**σ**” and “**d**” are the pooled standard deviation (SD) and difference of PVV (expressed as %) means of two groups. Given the pioneer character of our study, these two values were obtained from a previous study **[21]** aiming to evaluate the effects of two interval training programs of varying intensities (100% vs. 110% of maximal aerobic velocity [MAV]) on hematocrit, hemoglobin, and PVV in 39 young men. In the above-mentioned study participants were assigned to two control groups (CG_100_ (n = 9); CG_110_ (n = 10)), and two training groups (one with 100% MAV (TG_100_, n = 10) and one with 110% MAV (TG_110_, n = 10)) **[21]**. All participants accomplished a maximal graded exercise test, and an intermittent exercise protocol **[21]**. Blood samples were collected at rest, at the end of the intermittent exercise, and after 15 min of recovery, before and after 8-weeks-training **[21]**. The interval training sessions consist of 30s intermittent exercise run at 100% or 110% MAV with 30s recovery at 50% MAV **[21]**. One of the main results of the aforementioned study was that the TG_110_ and CG_110_ have similar mean values of post-test (***i*.*e*.** after training) PVV_end_ (%) (***i*.*e*.** PVV measured at the end of the intermittent exercise): -8.4±2.1 vs. -11.1±3.8% **[21]**. The injection of the aforementioned data into the formula results in a total sample of 28 participants (14 in each group).

### Experimental design

All participants went to the laboratory (ISSEP Ksar-Said of Tunisia) for a familiarization session with all of the experiment’s materials and procedures. Each participant came to the research facility pre- and post- program for a medical assessment and anthropometric measurements performed by an anthropometric evaluator (*NG in the authors’ list*). CG did not participate in any physical training program during this experimental period, but they continued their regular practice of physical activity (***e*.*g*.** handball, football, swimming) at the Institute of Sports and Physical Education.

**Fig 1:**
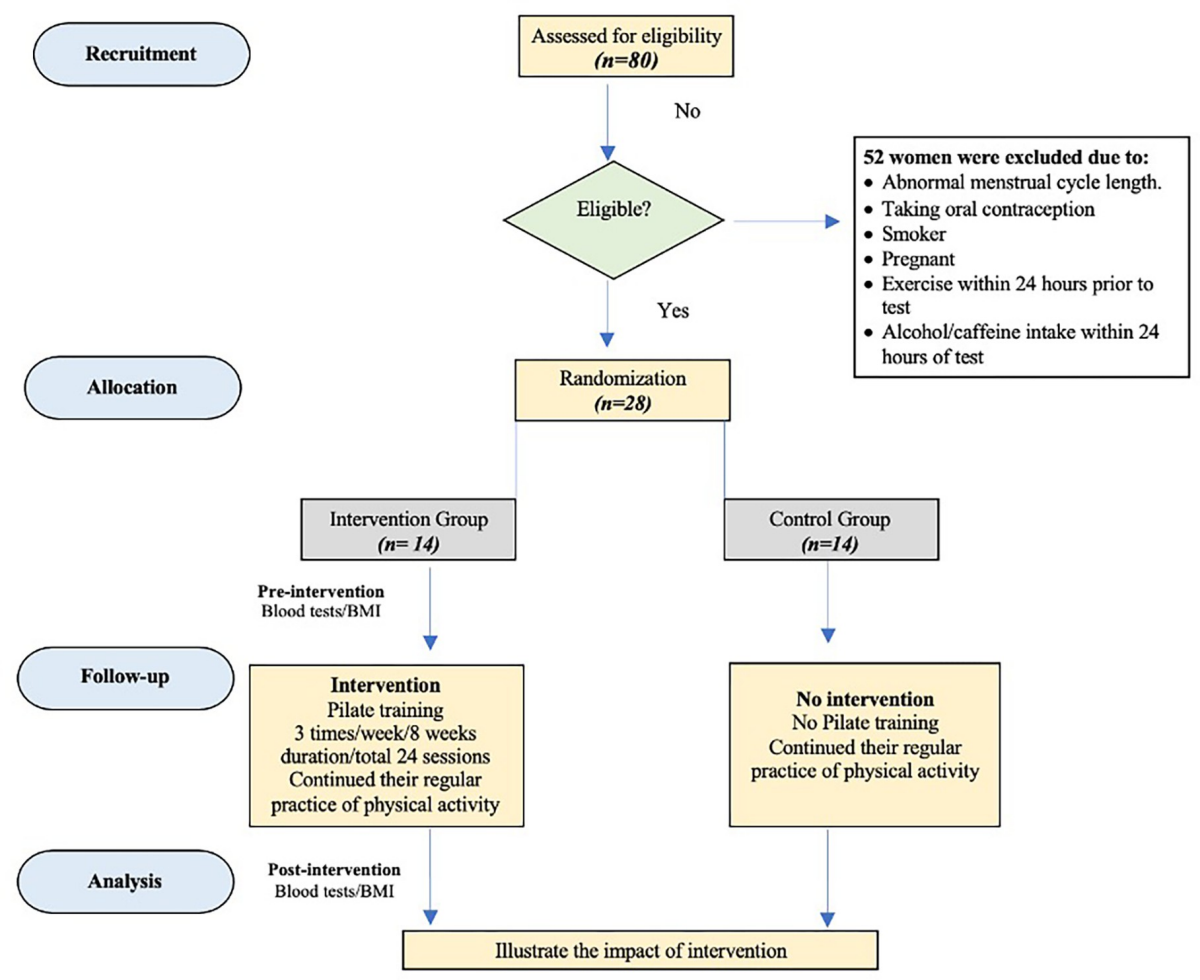
Flowchart.

The menstrual cycle is divided into three phases, as previously described by Ghazel et al. **[23]**. These phases are menstrual phase, follicular phase, and luteal phase. Women experienced pain both before and during their menstrual periods **[23]**. All tests were carried out during the menstrual phase. All tests were completed within two weeks, both before- and after- PT in thermoneutral condition **[24, 25]**. All sessions were performed at the same time of the day to minimize the effects of diurnal variation in the measured parameters **[26]**. **Fig 1** outlines the study protocol.

### Pilates training program

For eight weeks, the PT group participated in a PT program 3 times/week (24 sessions in total). The intervals between testing sessions for recovery were at least equal to 48 hours. PT sessions included: ***(i)*** a standardized warm-up of five minutes of continuous jogging, followed by five minutes of dynamic exercises (***e*.*g*.** high knee skip, high knee run, heel kick), ***(ii)*** a training session (**Table 2**), and ***(iii)*** a cool-down of approximately five minutes of static stretching.

**Table 2:** Pilates training program sessions’ exercise during 8 weeks.

Exercise	Week 1	Week 2	Week 3	Week 4	Week 5	Week 6	Week 7	Week 8
**Hundred**	10 cyc / 10 batt	10 cyc / 10 batt	10 cyc / 10 batt	10 cyc / 10 batt	10 cyc / 10 batt (flexed leg at 90°)	10 cyc / 10 batt (flexed leg at 90°)	10 cyc / 10 batt (flexed leg at 90°)	10 cyc / 10 batt (flexed leg at 90°)
**Shoulder Bridge (raising/ lowering the pelvis)**	12 Rep	12 Rep	12 Rep	12 Rep	12 Rep	12 Rep	12 Rep	12 Rep
**Shoulder Bridge (pelvis up + leg kicks)**	-	-	-	-	6 Rep for each leg	6 Rep for each leg	6 Rep for each leg	6 Rep for each leg
**One leg stretch**	10 Rep for each leg	10 Rep for each leg	10 Rep for each leg	10 Rep for each leg	10 Rep for each leg	10 Rep for each leg	10 Rep for each leg	10 Rep for each leg
**Double Leg stretch**	-	-	-	-	10 Rep	10 Rep	10 Rep	10 Rep
**One leg circle**	-	-	-	-	6 Rep for each side / leg	6 Rep for each side / leg	8 Rep for each side / leg	8 Rep for each side / leg
**Roll up Roll down**	10 Rep	10 Rep	10 Rep	10 Rep	10 Rep (leg stretched)	10 Rep (leg stretched)	**replace with Neck pull: 8 Rep**	**replace with Neck pull: 8 Rep**
**Rolling like a ball**	10 Rep	10 Rep	10 Rep	10 Rep	10 Rep	10 Rep	10 Rep	10 Rep
**Open leg rocker **	10 Rep (alternating mvt)	10 Rep (alternating mvt)	10 Rep (alternating mvt) 10 Rep (simultaneous mvt)	10 Rep (alternating mvt) 10 Rep (simultaneous mvt)	8 Rep (simultaneous mvt)	8 Rep (simultaneous mvt)	8 Rep (simultaneous mvt)	8 Rep (simultaneous mvt)
**Side kick**	-	-	8 Rep Up down 8 Rep Front back (for each side)	8 Rep Up down 8 Rep Front back (for each side)	8 Rep Up down 8 Rep Front back (for each side)	8 Rep Up down 8 Rep Front back (for each side) 8 rotations (for each side)	8 Rep Up down 8 Rep Front back (for each side) 8 rotations (for each side)	8 Rep Up down 8 Rep Front back (for each side) 8 rotations (for each side)
**Criss cross**	-	-	8 Rep for each leg	8 Rep for each leg	10 Rep for each leg	10 Rep for each leg	10 Rep for each leg	10 Rep for each leg
**Spine Twist**	6 Rep for each side	6 Rep for each side	6 Rep for each side	6 Rep for each side	6 Rep for each side	6 Rep for each side	6 Rep for each side	6 Rep for each side
**Spine strech Forward**	6 Rep	6 Rep	8 Rep	8 Rep	6 Rep (leg stretched)	6 Rep (leg stretched)	8 Rep (leg stretched)	8 Rep (leg stretched)
**Knee push up**	-	-	-	-	-	-	6 Rep	8 Rep
**Plank**	-	-	30 ‘‘	60 ‘‘	60 ‘‘	60 ‘‘	60 ‘‘	60 ‘‘
**Swimming**	10 Rep (slow rhythm)	10 Rep (slow rhythm)	12 Rep (fast rhythm)	12 Rep (fast rhythm)	10 Rep (fast rhythm)	10 Rep (fast rhythm)	12 Rep (fast rhythm)	12 Rep (fast rhythm)

**Cyc**: cycle; **mvt**: movement;.**rep**: repetition.

### Anthropometrics parameters

Using an electronic scale, body mass was measured to the nearest 0.1 kg with the participant wearing light clothing and no shoes (Kern, MFB 150K100). A measuring tape fixed to the wall was used to determine height to the nearest 0.5 cm. Body mass index (BMI, kg/m^2^) was calculated. All measurements were taken by the same evaluator (*NG in the authors’ list*) in accordance with the International Biological Program’s positions and techniques **[27]**.

### Blood analyses

Sample collection (15 mL) occurred before the beginning and after PT, when the participants were at rest and awake. A heparinized catheter was inserted into an antecubital vein to collect a venous blood sample. Blood was collected in a vacutainer tube containing anticoagulant Tetra Acetic Diamine Ethylene Acid (EDTA). The EDTA was used to determine hematological parameters, ***i*.*e***. white blood cell (WBC), neutrophils, monocyte, lymphocyte, hemoglobin, and hematocrit. In general, hematological parameters were measured in a multichannel automated Hematology Analyzer Sysmex XS-1000i within three hours. Determinations of both hematocrit and hemoglobin were performed to estimate PVV according to the method developed by Dill and Costill **[28]**:

$$PVV\;(\%)=100\;x\;(\frac{Hemoglobin\;(\frac{g}{dl})1}{Hemoglobin\;(\frac{g}{dl})2}\times \frac{\left(1-Hematocrit\;(\%)2\times 10^{-2}\right)}{\left(1-Hematocrit\;(\%)1\times 10^{-2}\right)})‐100$$

(PVV) is expressed as % PVV, ***1***: value measured before PT program, **2**: value measured after PT program.

### Statistical analyses

All the statistical analyses were performed using SPSS version 23.0 for Windows (SPSS, Inc., Chicago, IL, United States). However, before conducting such analyses, the normality of distributions was tested with Shapiro-Wilk’s test. Shapiro-Wilk’s result test was not significant (p > 0.05). Data were analyzed using analysis of variance with repeated measures (periods × groups). When necessary, the Bonferroni post hoc was applied to identify significant differences after confirming significant group differences over time. An independent samples t-test was performed for PVV to compare the difference between TG and CG values. Correlation analysis was performed by a Pearson’s correlation. Effect sizes were calculated as partial eta-squared (η_p_^2^) with values ≤ 0.010 expressing a trivial effect; values between 0.010 and 0.059 expressing a small effect; values between 0.060 and 0.139 a moderate effect, and values ≥ 0.140 a large effect **[29]**. Additionally, the (*d)* was calculated for pairwise comparison according to Cohen **[29]**. The magnitude of (*d*) was interpreted as trivial (< 0.2), small (0.20 to 0.49), moderate (0.50 to 0.79), large (≥ 0.80) **[30]**. The level of significance was predetermined to be p < 0.05 for all statistical analyses. The percentage of variation (%) of all variables was also calculated to establish changes between pre- and post- tests using the following formula:

$$\left(\frac{Final\;value-Initial\;value}{Initial\;value}\right)\times 100$$

## Results

### Hematological parameters and plasma volume

Mean ± SD values for selected hematological parameters measured before- and after- PT are presented in **Table 3**. There was a significant “main effect” of period on neutrophils (p = 0.034, η_p_^2^ = 0.46), WBC (p = 0.037, η_p_^2^ = 0.27), hemoglobin (p = 0.018, η_p_^2^ = 0.49) and hematocrit (p = 0.009, η_p_^2^ = 0.51). However, there was no significant “main effect” of period on monocyte (p = 0.134, η_p_^2^ = 0.24) and lymphocyte (p = 0.739, η_p_^2^ = 0.01). A significant (periods × groups) interaction was obtained for neutrophils (p = 0.004, η_p_^2^ = 0.62), WBC (p = 0.022, η_p_^2^ = 0.33), hemoglobin (p = 0.027, η_p_^2^ = 0.34), and hematocrit (p = 0.021, η_p_^2^ = 0.39). No significant (periods × groups) interaction was obtained for monocyte (p = 0.214, η_p_^2^ = 0.06) and lymphocyte (p = 0.433, η_p_^2^ = 0.08). Analysis revealed lower values of neutrophils (32%, p = 0.01), WBC (19.4%, p = 0.01), hemoglobin (4.6%, p < 0.05), and hematocrit (4.3%, p < 0.05) in PG as compared to CG.

**Table 3:** Effects of 8 weeks of Pilates training program on hematological parameters in both Pilates training (PG) and control (CG) groups.

	PG (n = 14)		CG (n = 14)		p (η_p_^2^)		
	Pre-test	Post-test	Pre-test	Post-test	Interaction:group x period	Main effect:period	Main effect:group
**WBC (10** ^**9**^ **/L)**	7.21±1.5	5.98±1.3	7.40±1.7	7.30±1.3	0.022 (0.33)	0.037 (0.27)	0.153 (0.15)
**NE (10** ^**9**^ **/L)**	3.95±1.1	3.04±0.8	4.13±0.9	3.94±1.1	0.004 (0.62)	0.034 (0.46)	0.152 (0.36)
**MO (10** ^**9**^ **/L)**	0.54±0.1	0.50±0.1	0.60±0.1	0.60±0.2	0.214 (0.06)	0.134 (0.24)	0.156 (0.21)
**LY (10** ^**9**^ **/L)**	2.51±0.4	2.13±0.3	2.69±0.8	2.57±0.7	0.433 (0.08)	0.739 (0.01)	0.167 (0.22)
**Ht (%)**	39.92±2.2	37.68±2.4	40.58±3.4	39.79±3.0	0.021 (0.39)	0.009 (0.51)	0.732 (0.02)
**Hb (g/dl)**	12.96±0.8	12.23±0.9	13.14±1.0	12.91±1.1	0.027 (0.34)	0.018 (0.49)	0.605 (0.03)

Data were mean values (± SD).

**Hb**: hemoglobin, **Ht**: hematocrit, **LY**: lymphocytes, **MO**: monocytes, **NE**: neutrophils, **p (η**_**p**_^**2**^**)**: probability (partial eta-squared), **PVV**: plasma volume variation, **WBC**: white blood cells.

**Fig 2:**
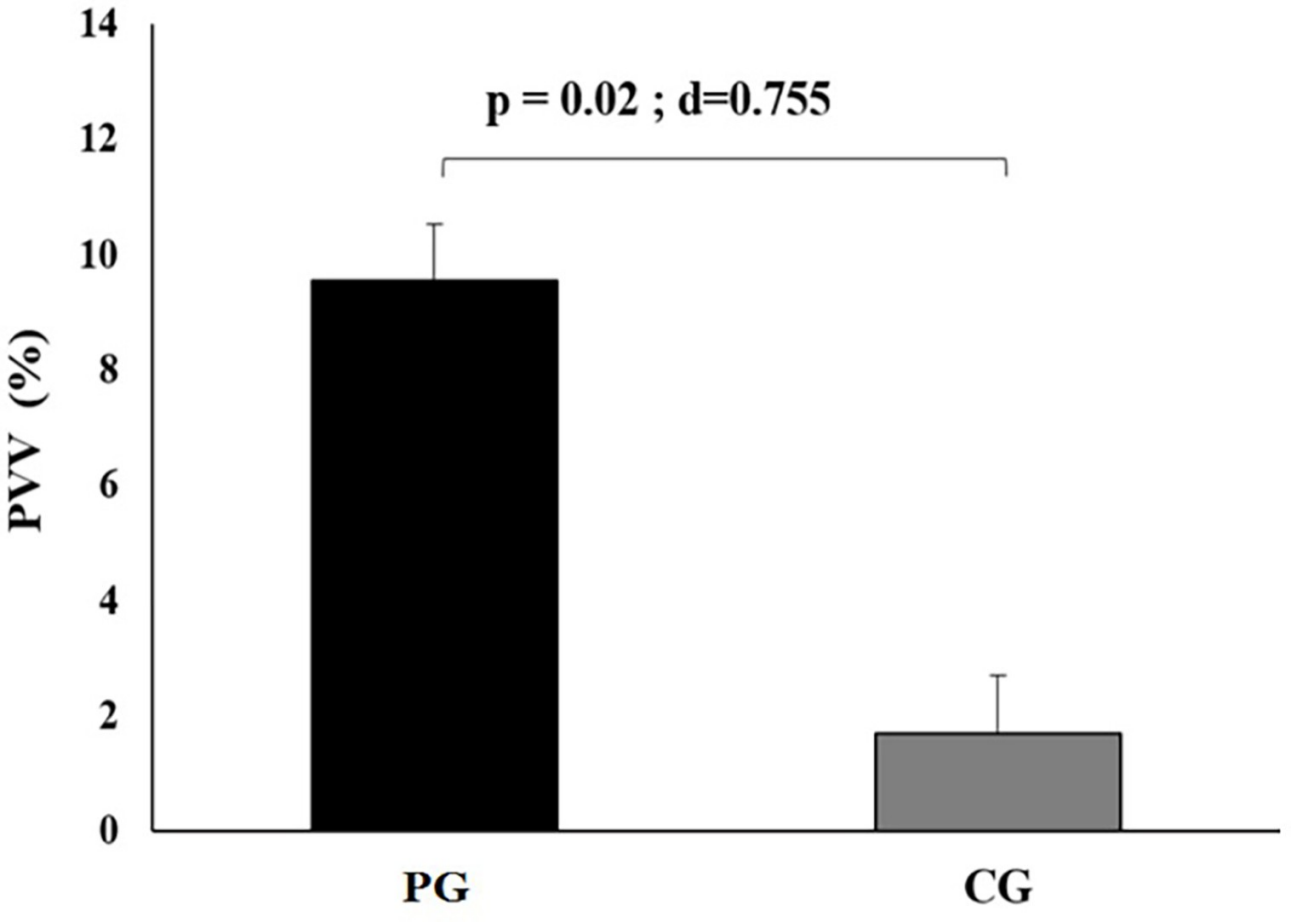
Plasma volume variation (PVV) in response to Pilates training program in both Pilates training (PG, n = 14) and control groups (CG, n = 14).

The changes in PVV after eight weeks under both conditions are presented in **Fig 2**. PVV was significantly higher in PG compared to CG (p = 0.02, d = 0.75).

## Discussion

To the best of the authors’ knowledge, this is the first study to look into the impact of PT on hematological parameters and PVV. The current study reported that hematocrit and hemoglobin levels decreased in response to the PT program. These changes resulted in a significant increase in PVV following PT program. These findings are consistent with those of Ben Abderrahman et al. **[31]** and Rhibi et al. **[21]**, who reported a significant reduction in hematocrit and hemoglobin at rest following a high-intensity interval training program. Rhibi et al. **[21]** explained their results by figuring out that the plasma volume increase limits the hemoglobin and hematocrit variations because they exhibited a significant increase in PVV after the training program. Alternatively, the reduction in hematocrit in response to endurance training could be due to a disproportional rise in red blood cells compared to an increase in plasma volume **[32]**. Furthermore, the accumulation of some metabolites such as lactate ions, ammonium, and potassium in affected working muscle may enhance the intracellular osmotic pressure which leads to an efflux of water from blood space to an intracellular one **[33]**.

Our findings revealed that the PT program resulted in a small decrease in WBC and neutrophils. Contrarily, no significant differences in monocyte and lymphocyte were found following the PT program. Indeed, high total WBC, neutrophils, and lymphocyte counts were attributed to lower fitness level and obesity **[34]**. As a result, an elevated WBC level is associated with an increased risk of morbidity and mortality rates from coronary heart disease **[34]**. While acute exercise bouts have been linked to an increased inflammatory state **[35, 36]**, greater levels of engagement in physical activity have been linked to decreased systemic inflammation, and aerobic exercise training has also been shown to lower WBC counts **[37]**. Our findings are consistent with the findings of Node et al. **[37]**, Johannsen et al. **[34]**, and Sellami et al. **[38]**, who reported that aerobic exercise is linked to lower overall WBC and neutrophils counts. Furthermore, physical exercise is a non-pharmacological strategy that is suggested to combat the age-related decline in immunity with no negative side effects **[39]**. Evidence supported that PT improved symptoms of depression, anxiety, and mental health outcomes **[40]** that may be affected during the menstrual phase **[23]**. We suggest therefore that the enhancement of the immune system after PT could be related to positive emotional states induced by eight weeks of PT.

Our results suggest that eight weeks of mat PT reinforce the immune system and enhances the fluid regulatory. The current finding could enable physicians, coaches, and practitioners to take action by using PT program in order to enhance physical performances. Interestingly, PT could potentially help to counteract the negative effects of isolation and confinement stress on immune competency. Taking into consideration the coronavirus disease 2019 (COVID-19) pandemic, application of such training program could be helpful in boosting the immune system and stay active. In addition to its role as improving the immune system, PT could be practiced at home and thus contributes at having low risk of contracting COVID-19 while training in gym.

The present study has some limitations. First, Dill and Costill equation **[28]** was used for the first time for acute changes in plasma volume and not for chronic exercise. Second, the catecholamines was not assessed, which could provide a better understanding on the effects of PT on biochemical response. Third, the study was conducted exclusively in young active women, and data cannot be extrapolated to inactive women.

## Conclusions

The current results demonstrated clearly that mat PT led to a significant increase in PVV, and decrease in WBC and neutrophils, that could be often accompanied by an improvement in immune function.

## Supporting information

S1 File Data of the 28 participants. (XLSX)
